# Disease modelling with *in vitro* vascularised organoids

**DOI:** 10.1242/dmm.052769

**Published:** 2026-05-20

**Authors:** Chloe Kan, Lu Liu, Rio Sugimura

**Affiliations:** ^1^ School of Biomedical Sciences, Li Ka Shing Faculty of Medicine, The University of Hong Kong, Hong Kong SAR, China; ^2^ InnoHK Centre for Translational Stem Cell Biology, Hong Kong Science Park, Hong Kong SAR, China

**Keywords:** Vascularised organoids, Disease modelling, Assembloids, Co-differentiation, Organ-on-a-chip

## Abstract

Organoids are 3D systems derived from stem cells that recapitulate human tissue microenvironments, offering a useful tool for a wide range of investigations, including developmental biology, drug screening and disease modelling. As the vasculature is critical in biological processes within the human body, both in health and disease, recent efforts in the field have focused on developing vascularised organoids. In this At a Glance article, we discuss a number of currently available vasculature fabrication approaches, including co-culture with vascular endothelial cells, assembloids, co-differentiation, genetic engineering, 3D bioprinting and organ-on-a-chip systems, along with their advantages and limitations. We also provide a comparison of these fabrication approaches and their use for modelling diseases. Finally, we discuss current challenges and future directions regarding the use of vascularised organoids to mimic human physiological conditions and as clinically relevant models of human diseases.

## Teaser

**Summary:** This At a Glance article discusses current strategies for vascularising organoids, highlighting how different approaches can enhance physiological relevance and translational potential in disease modelling.

**Figure:**
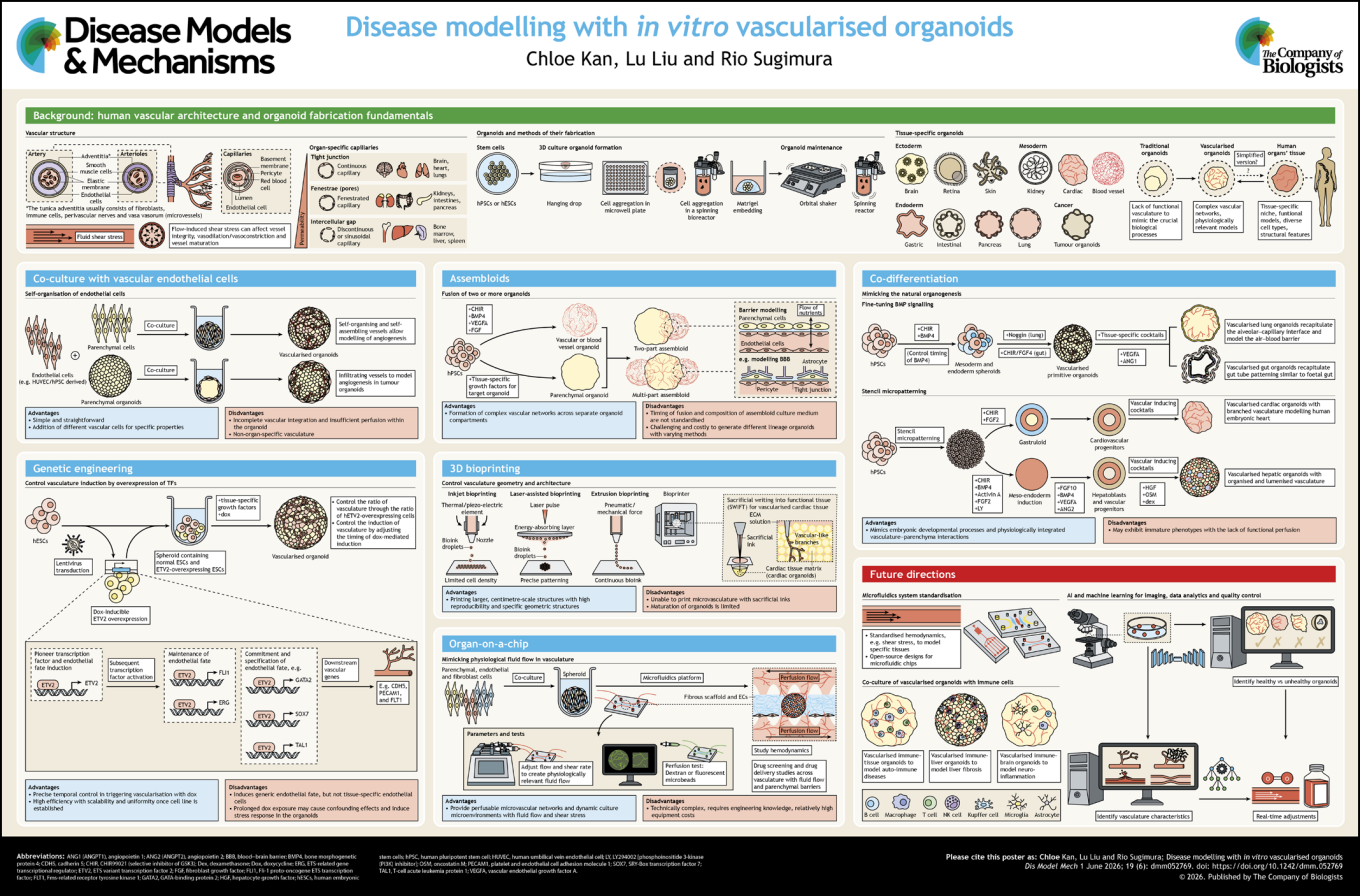


## Introduction

More than a decade has passed since the first reported three-dimensional (3D) organoid system was derived from stem cells (Corrò et al., 2020; Sato et al., 2009). Since then, organoids have revolutionised *in vitro* disease modelling by enabling the fabrication of structures that resemble human organs. For example, kidney organoids with self-organising nephron structures (Takasato et al., 2014), liver organoids with a functional bile canaliculi network (Ramli et al., 2020) and cortical organoids mimicking the neocortex layers of the mammalian brain (Kadoshima et al., 2013). These organoid systems bridge the gap between two-dimensional (2D) human-based culture models and animal models that feature 3D organ architecture and cellular diversity. Traditional 2D monolayer cultures fail to capture the complex tissue-like architecture of cells, while animal models are limited by genetic differences that cause variations compared with human physiology. By using human cells to create 3D *in vitro* systems with self-organising structures, organoids are not limited to monolayer growth and recapitulate native tissue architecture, multicellular interactions and physiological functions. Organoid technology has advanced rapidly in recent years from creating single-lineage organoids to incorporating multi-lineage organoids and cell types in a single system, offering a more physiologically relevant model of human tissues. For instance, fusing organoids of different brain regions can model cell migration and regional interactions (Bagley et al., 2017), while fusing spinal and muscle organoids can model neuromuscular diseases (Faustino Martins et al., 2020). Despite this, several major limitations have been identified in conventional organoid systems: limited size owing to necrotic cores caused by poor nutrient diffusion, difficulties in scalability and reproducibility, and the lack of functional vasculature to mimic crucial biological processes in the human body.

In this At a Glance article, we focus on current approaches to the fabrication of vascularised organoids. The absence of vascular networks limits organoids to millimetre-scale sizes, restricting the volume, complexity and functional maturity of organoid tissues. The lack of vasculature causes inadequate nutrient and oxygen delivery to the inner layers of organoids, which can lead to necrotic cores due to nutrient deprivation and hypoxia (Chiew et al., 2017; Shin et al., 2025b). The resulting oxygen shortage is often compensated for by a hyperoxic environment (i.e. atmospheric oxygen levels of ∼21%) in standard culture incubators, which fails to mimic physioxia conditions of human tissues, i.e. oxygen levels of 2−10% (Carreau et al., 2011; Umans and Gilad, 2025). Therefore, introducing vasculature into organoids can ensure dynamic regulation of oxygen supply and support tissue growth, better resembling native organs for disease modelling.

In addition to maintaining organoid viability, vasculature plays a key role in tissue homeostasis and pathophysiology. Vascular networks are essential for intercellular communications, particularly facilitating the transport of signalling molecules and immune cells in a tissue-specific manner (O'Connor et al., 2022), further mediating tissue development and disease progression. For example, cardiovascular disease can result directly from endothelial cell dysfunction and inflammation, disrupting endothelial-dependent vasodilation (Sun et al., 2020). Similarly, neurodegenerative diseases can involve vascular abnormalities, leading to impaired blood−brain barrier (BBB) integrity, neuroinflammation and damage to brain tissues (Mekala and Qiu, 2025; O'Connor et al., 2022). Therefore, incorporating vasculature into organoids does not only improve their survival but also allows the development of physiologically relevant models in order to study vascular−parenchymal interactions, drug responses across tissues and personalised medicine applications.

Introducing vasculature into organoids has long been a focus of the field of research and in this At a Glance article we discuss recent advances, such as co-development of mesoderm-endoderm lineages, genetically inducible endothelial niches and microfluidics technology that, together, form a toolkit of possible approaches to vascularised organoids. Given the rapid development of complex organoid systems and the expanding applications of organoids in disease modelling, this At a Glance article summarises current vascularisation strategies, their physiological relevance and future challenges.

## Structural features and design criteria for vascularised organoids

The human vascular system comprises endothelial cells (ECs) that line the inner lumen of vessels and is further supported by mural cells (pericytes and smooth muscle cells) (Siekmann, 2023). Capillaries consist of a pericyte-lined tube of ECs, forming continuous, fenestrated or discontinuous/sinusoidal structures (Gomez-Salinero et al., 2025), while larger vessels contain ECs surrounded by smooth muscle cells (Lin et al., 2021) (see poster ‘Background: human vascular architecture and organoid fabrication fundamentals’). Mural cells are crucial for regulating vascular tone and perfusion, and for controlling vasoconstriction and vasodilation via signals from ECs (Dunaway et al., 2025). Vascular characteristics are also affected by hemodynamics, such as flow-dependent shear stress (Bertani et al., 2023), which has been shown to regulate vessel maturation (Goettsch et al., 2008). The complex vascular ecosystem not only enables nutrient and oxygen exchange but also establishes tissue-specific vascular niches. For instance, single-cell analysis of 19 human organs and tissues identified 42 vascular populations with organ-specific ECs specialisations (Barnett et al., 2024). Therefore, replicating vasculature across tissues by using organoid systems is critical to advance understanding of development and disease modelling (Shin et al., 2025a).

*In vitro* vasculature should be able to mimic perfusable networks with lumens and physiological permeability. Fluid perfusion is critical in driving vessel growth (Vila Cuenca et al., 2025), maintaining vascular structural integrity (Zhou et al., 2025) and nutrient delivery to parenchymal tissues for extended culture durations (Garreta et al., 2021). Optimal vascularised organoids should have a close cellular arrangement of vasculature and parenchyma, organ-specific EC functions, and *in vivo*-like hemodynamics (Shin et al., 2025a). Different principles can be applied to generate vascularised organoids, but most approaches are heavily inspired by human vascular ontogeny, particularly vasculogenesis (*de novo* formation of blood vessels from endothelial progenitors) (Käßmeyer et al., 2009), and angiogenesis (growth of pre-existing vessels) (Carmeliet, 2003). Co-culture mimics angiogenesis since assembloids are inspired by tissue-tissue interactions, while co-differentiation is inspired by vasculogenesis. Recently, strategies have shifted towards bioengineering to overcome perfusion, organoid maturation and reproducibility limits. Genetic engineering was designed for precise induction timing of vasculogenesis, 3D bioprinting for defined geometry, and an organ-on-a-chip approach aims for fluid perfusion and shear stress. Choosing a fabrication technique involves balancing complexity, scalability, physiological relevance and technical feasibility. In the following section, we discuss the mechanistic details of each approach, with specific examples of successful applications.

## Applications of different fabrication methods of vascularised organoids

### Co-culture with vascular endothelial cells

Co-culturing organoids with exogenous vascular ECs (see poster ‘Co-culture with vascular endothelial cells’) is one of the earliest methods used to induce vascularisation, enabling angiogenesis and self-organisation through cell signalling. Human umbilical vein endothelial cells (HUVECs) are one of the most widely used ECs for angiogenesis due to their availability, simple culture conditions and tube formation abilities (Arnaoutova et al., 2009). However, HUVECs lack tissue-specificity and have limited microvasculature formation ability due to the absence of mural cells (Nwokoye and Abilez, 2024; Werschler et al., 2024). Mesenchymal stem cells (MSCs) are multipotent cells that can differentiate into mural cells to support vessel formation and maturation (Pill et al., 2015; Tian et al., 2017; van Dijk et al., 2015; Warren and Gerecht, 2023). Induced pluripotent stem cells (iPSCs) can be used to derive both ECs and mural cells (Orlova et al., 2014; Park et al., 2023), enabling patient-specific disease modelling (Paik et al., 2020).

Co-culturing organoids with vascular ECs is beneficial for cancer angiogenesis models. Inclusion of ECs in liver cancer organoids upregulates angiocrine factors, including MCP-1 (officially known as CCL2), IL8 and CXCL16 (Lim et al., 2022). MCP-1 recruits pro-angiogenic myeloid cells and ECs (Salcedo et al., 2000), leading to increased survival of tumours (Liu et al., 2020; Salcedo et al., 2000). IL8 promotes EC proliferation (Rizzo et al., 2022) and associates with tumour metastasis and extravasation (Meier and Brieger, 2025; Wen et al., 2020). CXCL16 regulates immune cell chemotaxis (Kim et al., 2019) and increases vascular EC density in cancer (Deng et al., 2024). These factors are crucial for recapitulating the tumour microenvironment, enabling the study of angiogenic pathways and anti-cancer therapies (Chiew et al., 2017).

Aside from cancer research, incorporating human MSCs and HUVECs with iPSC-derived hepatic endoderm cells demonstrated organogenetic interactions and self-organisation of 3D liver buds (Takebe et al., 2013). Unlike the previously mentioned co-culture system, this method co-cultures vascularised cells with parenchymal cells before the formation of embryoid bodies (see poster ‘Co-culture with vascular endothelial cells’). One advantage of this method is that endothelial networks establish internally rather than infiltrating from outside, allowing better vasculature integration with parenchymal cells, enhancing organoid core vascularisation and reducing necrosis. Similarly, Shi et al. co-cultured HUVECs with iPSCs to generate vascularised cerebral organoids that, compared to non-vascularised organoids, show reduced hypoxic cells, increased organoid sizes and thicker neuroepithelial layers (Shi et al., 2020). Patch-clamp recordings also showed that the vascularisation increases outward current amplitude (Shi et al., 2020), a key characteristic of cortical neuron maturation (Guan et al., 2011). In cardiac organoid studies, Voges et al. co-cultured cardiomyocytes with PSC-derived fibroblasts, ECs and pericytes, in a ratio similar to that of the human foetal heart (Sim et al., 2021; Voges et al., 2023). Vasculature improved the organoid contractile force to a similar rate as that in human heart muscles (Lunkenheimer et al., 2004; Voges et al., 2023) and upregulated cardiac troponin-I expression, a marker for cardiomyocyte maturation (Bedada et al., 2014). Therefore, vasculature not only facilitates nutrient transport but also improves the maturation of surrounding parenchymal cells, ultimately improving their functions and creating more physiologically relevant models.

One main advantage of the co-culture system is that multiple cell types can be easily incorporated into the organoid system. For example, pericytes can be added along ECs to support the formation of microvasculature (Li et al., 2025). However, transcriptional profiling and RNA-sequencing studies indicate that ECs exhibit heterogeneity across organs (Jambusaria et al., 2020; Marcu et al., 2018; Wakabayashi and Naito, 2023), suggesting that non-organ-specific vasculature may not support organ-specific maturation and barrier functions. This is evident when co-culturing of HUVECs with breast cancer cells did not form vasculature, but co-culturing of HUVECs with liver cancer spheroids formed disordered vascular networks and abnormal branching (Chiew et al., 2017). This lineage-dependent, organ-specific EC behaviour limits reproducibility across organoids. Whether commercial cell lines or iPSC-derived cells, researchers should select the most suitable, organ-matched EC sources to optimise co-culture outcomes for vascularised organoids.

### Assembloids: multi-organoid fusion

Recognising the complexity of tissue interactions, organoid technology has now moved beyond relying on single-organoid systems and co-culturing with single cells. Multi-lineage organoid systems can be created by co-culturing two organoids and enabling their fusion and the formation of so-called assembloids (see poster ‘Assembloids’). While fusion of multi-lineage organoids has been applied in various tissues, such as those of intestinal (Workman et al., 2017) and foregut−midgut (Koike et al., 2019), it is most established for neural organoids, such as cortical-motor (Andersen et al., 2020) and cortico-striatal assembloids (Miura et al., 2020). Recently, blood vessel organoids or vascular organoids have been created to mimic human vascular networks in 3D (Gong et al., 2025; Wimmer et al., 2019). These vascular organoids recapitulate human microvasculature networks with perfusable vasculature post-transplantation into mouse models (Gong et al., 2025; Wimmer et al., 2019). Fusing vascular organoids with parenchymal organoids can yield vascularised systems, such as brain-vascular assembloids exhibiting functional BBB-like structures (Sun et al., 2022). Moreover, tissue-region-specific fusions can model diseases effectively. For instance, cortical−vascular assembloids display Alzheimer's disease phenotypes (Kong et al., 2023), while neural−mesodermal assembloids explore peripheral nervous system development (Rockel et al., 2023). Recently, vascular immune organoids incorporating macrophages (Chau et al., 2024) have enabled the possibility of fabricating immune−vascular−parenchymal interactions for studying immune cell trafficking, inflammatory responses and toxicity screenings, including cytokine release syndrome and vascular leakage, which are important in drug testing and safety profiling.

Another important variable to consider is the fusion timing, as studies suggest that early fusion facilitates penetration of vasculature into the organ-specific organoids, whereas later fusion allows sufficient time for cell maturation. For example, Dao et al. fused day 75 cerebral organoids (with astrocyte induction) and day 15 vascular organoids (Dao et al., 2024), promoting astrocytic maturation and expression of tight-junction proteins that are crucial in modelling the BBB. However, in other studies, researchers prefer to use early-stage organoids for fusion. For example, Sun et al. fused brain organoids and vascular organoids on day 12 of culture (Sun et al., 2022), which improved vascular penetration in the smaller-sized organoids and prevented a necrotic core. Earlier fusion also enables crosstalk between ECs and organ-specific cells, thereby facilitating faster organoid maturation and cellular differentiation (Pham et al., 2018; Yip et al., 2023). Therefore, depending on the objective of an experiment, the timing of fusion should be carefully chosen, specifically considering whether prior differentiation of specific cell types before vascular introduction is required. Interestingly, Sun et al. also showed that brain organoids can be ‘sandwiched’ between two vascular organoids (Sun et al., 2022) (see poster ‘Assembloids’). Due to the increased area of invasion, this method allows a larger area of vascularisation along the two sides of the parenchymal organoid and, in the case of smaller-sized organoids, can achieve complete vascular coverage, which may be more important when modelling vascular diseases.

3D organoids require time to aggregate, differentiate and grow, which demands experienced personnel and frequent medium changes to prevent necrosis. Sophisticated reagents are often needed for culturing organoids. For example, basement membrane matrices for embedding can be costly and have batch-to-batch variability (Huang et al., 2021; Sisakht et al., 2025). Various growth factors in the culture medium can also increase expenses for long-term maturation and large-scale applications (Urbischek et al., 2019). The recipe of culture medium presents further complications in assembloid models, as the medium should cater for both parenchymal and vascular organoids. For instance, one study used brain organoid medium with the addition of vascular endothelial growth factor A (VEGFA) to maintain a vascular-brain assembloid (Sun et al., 2022), while another study used a mix of cortical organoid medium and vascular organoid medium at a 9:1 ratio for a vascular−cortical assembloid (Kong et al., 2023). The lack of standardisation of protocols, such as the exact duration of organoid culture until fusion and the composition of culture medium, hinders the ability of assembloid systems to contribute to high-throughput experiments (Zhou et al., 2023). In a clinical setting, patient-derived organoids have high potential in disease modelling and drug development. Yet, efforts are still needed to overcome challenges related to the speed of organoid development, cost and reproducibility (Bose et al., 2021).

### Co-differentiation: recapitulating developmental processes

The principle of co-differentiation is to mimic organogenesis, creating organoids with integrated vasculature that share the same origin as the parenchymal component (see poster ‘Co-differentiation’). Unlike co-culture with vascular ECs and assembloids, in which vascular and parenchymal tissues typically self-organise independently, co-differentiation allows their coordinated self-organisation, thereby establishing a vasculature-parenchymal crosstalk that more closely mimics natural development (Brassard and Lutolf, 2019; Holloway et al., 2019; Scott Baldwin, 1996). After gastrulation in embryonic development, the ectoderm forms the skin and brain (Jameson et al., 2023), the endoderm generates respiratory and gastrointestinal tracts (Nowotschin et al., 2019), while the mesoderm layer forms musculoskeletal and cardiovascular systems (Ferretti and Hadjantonakis, 2019), including ECs (Goldie et al., 2008; Stone and Stainier, 2019). Mesoderm induction by growth factors often uses the GSK3 inhibitor CHIR99021 (CHIR) for driving differentiation (Buikema et al., 2020) and bone morphogenetic protein 4 (BMP4) for epithelial−mesenchymal transition (Richter et al., 2014). Micropatterning and interactions between the germ layers are crucial for organ-specific ECs (Deglincerti et al., 2016; Warmflash et al., 2014). Therefore, strategies for co-differentiation use developmental cues as a guide and simultaneously induce cells from different lineages, generating organoids composed of diverse, developmentally linked cell types.

The co-differentiation approach is particularly important in tissues, such as lung and intestine, where vascular networks perform specialised functions. Previous attempts to fabricate lung organoids did not include vasculature and well-defined alveolar−capillary interfaces (Chen et al., 2017; Jacob et al., 2017; Miller et al., 2019), thereby failing to recapitulate the air−blood barrier, which is crucial for modelling lung diseases. Traditional methods for generating lung organoids often focus on endoderm induction (Ghaedi et al., 2015; Miller et al., 2019); however, a recent study deliberately altered the early differentiation conditions to create both endodermal and mesodermal lineages (Miao et al., 2025). Interestingly, the incorporation of CHIR and BMP4 in culture medium at days 0-1 of embryoid body differentiation promotes lung lineage specification, whereas sustained addition of BMP4 at days 0-3 promotes intestinal lineage specification (Miao et al., 2025). In terms of vasculature, the protocol described by Miao et al. only requires VEGFA and ANG1 (officially known as ANGPTL1) to promote vasculature maturation, avoiding the need for other angiogenic factors, such as those used in the previous protocol for vascularised intestinal organoids (Holloway et al., 2020). By fine-tuning BMP activation and selecting specific angiogenic factors, Miao et al. successfully generated vascularised lung and intestinal organoids consisting of organ-specific endothelial cells (see poster ‘Co-differentiation’), as validated by their respective organ-specific markers (Miao et al., 2025).

One limitation of the co-differentiation approach is that the resulting vascularised organoids are closer to the early embryonic and foetal developmental stage due to the early-stage differentiation of mesodermal and endodermal lineages. Although additional studies are needed to assess the suitability of these organoids for modelling adult diseases, this same characteristic makes co-differentiation models particularly suitable for studying early-stage development. For instance, due to ethical constraints on embryonic research, it is often difficult to directly study early vascularisation of the heart and liver in humans. Therefore, organoid systems are a crucial tool for modelling early vascularisation. In fact, recent studies have created vascularised cardiac (Lewis-Israeli et al., 2021) and liver (Harrison et al., 2023) organoids, enabling the investigation of organ vascular development (Abilez et al., 2025). Abilez et al. used micropatterning of human embryonic stem cells with CHIR and FGF2 to generate a gastruloid model, which was then differentiated into cardiovascular progenitors by using the inhibitor of Wnt response-1 (IWR-1) and vascularised by using a vascular factor cocktail (Abilez et al., 2025) (see poster ‘Co-differentiation’). The resulting vascularised cardiac organoid exhibited foetal-like beating and less variability than non-vascularised controls (Abilez et al., 2025). Applying the same angiogenic cocktail to mesendoderm lineages produced vascularised hepatic organoids with branched vasculature (Abilez et al., 2025) (see poster ‘Co-differentiation’). This suggests that standardised vascular factor combinations can be reused across organ types with a micropatterning and co-differentiation framework, thereby improving protocol reproducibility.

Co-differentiation emphasises balancing opposing signalling for mesoderm and endoderm. The protocol described by Miao et al. requires precise timing and careful dosage of BMP and Noggin (BMP antagonist) that involves extensive optimisation, and deviations can distort the patterning of the lineages (Miao et al., 2025). This complexity hinders transfer, scaling and adaptation across human pluripotent stem cell (hPSC) lines compared to co-culture or assembloid strategies. There is also limited control over vascular architecture and density, as vasculature emerge from intrinsic programs reliant on a cocktail of growth factors, as described by Abilez et al.’s work (Abilez et al., 2025) (see poster ‘Co-differentiation’). The use of complex variations of medium raises costs and limits the scalability that is required for drug screening or large-scale disease modelling. Simplifying protocols and standardising reagents is essential to ensure reproducibility.

### Genetic engineering: vascular modulation by using transcription factors

Genetic engineering reduces the reliance on growth factor-based differentiation and enables the precise timing of vasculature induction in organoids (see poster ‘Genetic Engineering’). Transcription factors can be modulated to promote vascular formation by regulating the expression of genes essential for vasculogenesis and angiogenesis. The formation of vasculature depends on multiple signalling pathways via major regulating transcription factors, including ETS variant transcription factor 2 (ETV2) and members of the GATA and SOX families (Bolte et al., 2018; Gomez-Salinero et al., 2025; Payne et al., 2024). In particular, ETV2 has been shown to drive hematopoietic and endothelial lineages (Gong et al., 2022; Koyano-Nakagawa et al., 2012). *ETV2* overexpression allowed the reprogramming of human fibroblasts into functional ECs (Lee et al., 2017) and the differentiation of hPSCs into brain-specific ECs with an increase in tight junction proteins (Ding et al., 2025). These lineage and differentiation studies established a foundation for engineering hETV2-overexpressing ECs to create vasculature in 3D organoid systems.

By designing a doxycycline (dox)-inducible system of tetracycline-controlled transcriptional activation, the timing for *ETV2* overexpression and vascular induction can be easily controlled. At the same time, the ratio of vasculature and parenchymal cells can also be optimised. Cakir et al. found that 20% *ETV2*-overexpressing cells with continuous dox-induction from day 18 efficiently generates vascularised human cortical organoids with high levels of EC markers and BBB characteristics, and accelerates neuronal maturation compared to non-vascularised cortical organoids (Cakir et al., 2019). Similarly, Maggiore et al. used ETV2-overexpressing iPSCs to generate kidney organoids and exposed the organoids to dox on days 5-18 of culture to generate endothelial lineages, creating vascularised human kidney organoids with mature nephrons (Maggiore et al., 2024). This optimised timing and duration of dox-mediated ETV2 activation minimised the off-target effects of dox and preserved maturation of nephron structures, podocytes and renin-positive cells without exogenous stimuli (Maggiore et al., 2024). More recently, Gong et al. used dox-inducible expression of *ETV2* for ECs (Gong et al., 2022) and *NKX3-1* for perivascular mural cells (Carson et al., 2000) to create vascular organoids (Gong et al., 2025). These genetic engineering approaches achieved vascularisation and induction of ECs without additional growth factors, such as VEGFs, which was often thought to be essential for creating ECs (Apte et al., 2019; Augustin and Koh, 2024; Ham et al., 2020; Jain, 2003).

Genetic engineering offers a simpler alternative to the complex growth factor timing during co-differentiation (see poster ‘Genetic Engineering’). Inducible transcription factors provide a more-straightforward route to vascularisation with precise temporal control via dox, which cannot be achieved in co-culture, assembloids or during co-differentiation. However, genetic engineering requires stable integration of transgenes, and the use of viral vectors risks off-target effects (Zhang et al., 2025) and raises ethical and/or safety concerns for translation (Munsie and Gyngell, 2018). Furthermore, all the discussed vascularisation methods lack continuous fluid flow and shear stress, both of which are crucial for the maturation of ECs (Cheng et al., 2025; Nwokoye and Abilez, 2024). Applications that require more physiologically relevant vasculature may require other bioengineering approaches.

### 3D bioprinting

The timing of vascular induction can be regulated using dox-inducible and genetic engineering approaches, but achieving spatial precision and systematically controlling vasculature architecture remains difficult. 3D bioprinting offers an alternative approach to creating organised and hierarchical vascular networks. Vascularised organoids can be generated by enabling the spatially controlled deposition of bioinks (cells and bio-compatible polymers) into specific architectures that support perfusable networks. Various bioprinting methods have emerged in recent years, including inkjet-based, laser-assisted and extrusion-based bioprinting. Inkjet-based bioprinting is a thermal or piezoelectric force-based approach, allowing droplet printing at high speed and low costs; however, it can only print limited cell densities (Ren et al., 2024). Laser-assisted bioprinting is a non-nozzle bioprinting method that allows high-precision patterning with only minimal shear stress on cells but risks photonic cell damage due to laser energy (Ventura, 2021; Vijayavenkataraman et al., 2018). Last, extrusion-based bioprinting relies on pneumatic or mechanical forces, creating a continuous flow of bioink for high scalability, although with relatively low resolution (Vijayavenkataraman et al., 2018) (see poster ‘3D bioprinting’). Currently, the most common bioprinting method is extrusion-based bioprinting (Tian et al., 2021), which enables printing larger, centimetre-scale structures with high reproducibility and specific geometric structures traditional organoid cultures cannot achieve (Nwokoye and Abilez, 2024) as, for example, highly reproducible kidney organoids (Lawlor et al., 2021), centimetre-scale intestinal organoids (Brassard et al., 2021) and chambered cardiac organoids (Kupfer et al., 2020). This lays the foundation for using 3D bioprinting to fabricate more-complex and geometrically relevant vascularised organoids.

Various 3D bioprinting studies have been done for vascularisation of tissues, such as dermal tissue (Baltazar et al., 2020), skeletal muscles (Choi et al., 2019) and hepatic lobules (Kang et al., 2020). 3D bioprinting can enable the vascularisation of organoids by using continuous extrusion-based methods, and sacrificial materials to construct vascular lumen structures for perfusion and fluid flow (Buckley et al., 2025; Huang et al., 2025; Miller et al., 2012). In particular, Skylar-Scott et al. developed a bioprinting strategy called sacrificial writing into functional tissue (SWIFT) that uses densely packed organoid matrices and sacrificial gelatine ink to embed vascular networks into tissues (Skylar-Scott et al., 2019) (see poster ‘3D bioprinting’). By leveraging the SWIFT technology, Skylar-Scott et al. replicated the geometry of arteries in the cardiac tissue and created perfusable vasculature with diagonal and septal branch-like structures in the matrix (Skylar-Scott et al., 2019). The cardiac organoids in the matrix eventually fuse, creating a large assembloid-like tissue structure with embedded vasculature, forming a perfusable cardiac tissue that can beat synchronously for over 7 days (Skylar-Scott et al., 2019). However, the SWIFT method only results in moderate contractility of the fused cardiac matrix compared to that of human adult cardiac tissues, and lacks microvasculature at capillary level (Skylar-Scott et al., 2019). Maturing organoids before forming the matrix and vasculature may be crucial for creating more mature, physiologically relevant models using SWIFT.

3D bioprinting for the fabrication of vascularised organoids is still relatively new and unexplored compared to other fabrication methods. Current sacrificial ink and extrusion-based printing are unable to print microvasculature structures that mimic capillaries with a small lumen (see poster ‘Vascular structure’). With further advancements of high-resolution printing techniques and bioink designs, 3D bioprinting has the potential to create vascularised organoids with more-defined vascular architectures.

### Organ-on-a-chip: dynamic perfusion

To realistically model human diseases, it is essential to model the dynamic microenvironment of tissues, including blood flow. Flow enables shear stress that regulates EC proliferation (Zhou et al., 2014), migration (Yue et al., 2025) and vessel maturation (Zhuang et al., 2025). For drug screening, perfusable vasculature is crucial for systemic exposure to assess absorption, metabolism, vascular toxicity (e.g. vessel leakage) and pharmacokinetics. Microfluidic technologies can create perfusable vessels and mimic physiologically relevant blood flow in vascularised organoids (see poster ‘Organ-on-chip).

In recent years, the organ-on-a-chip approach has been used to establish vasculature in various tissue types. In intestinal organ-on-a-chip models, perfusable vessels and continuous fluid flow allows the removal of necrotic cells from the growing epithelia (Nikolaev et al., 2020) and the spontaneous development of intestinal folds (Workman et al., 2018), enabling access to and better modelling of the intestinal lumen. Fluid travelling through the lumen can also be collected to analyse intestinal functions, such as nutrient digestion (Kasendra et al., 2018). In hepatic organ-on-a-chip models, fluid flow enhanced nutrient supply and waste exchange, inducing more-efficient cellular metabolism and promoting vascular−parenchymal interactions (Jin et al., 2018). In cardiac organ-on-a-chip, culture medium flow promotes EC integrity (Liu et al., 2024), more-mature sarcomeres (Di Cio et al., 2024) and increases contractility of cardiac tissues (Liu et al., 2025), enabling a more fitting model for cardiovascular diseases.

When modelling the human brain, one of the key features to consider is the BBB, which relies on tight junctions (Lochhead et al., 2020) and vascular permeability (Wu et al., 2023), and requires perfusion to recapitulate the appropriate microenvironment. Shear stress from perfusion induces the formation of tight junctions (Stanness et al., 1997), enhances BBB integrity (Cucullo et al., 2011), and affects BBB permeability and function (Shamul et al., 2025). Modelling of a functioning vasculature and BBB is especially relevant when modelling diseases – such as glioblastoma (Straehla et al., 2022) – where angiogenesis is crucial for the mechanism and pathology of the disease (Guarnaccia et al., 2018). Modelling the BBB may also create new platforms for drug screening, especially for testing the ability of drugs to cross the BBB (Park et al., 2019).

Due to multi-well formats and the ability to systematise flow conditions across chips, using microfluidics to generate vascularised organoids is advantageous for high-throughput drug screening. Furthermore, due to modular chip designs and interconnected fluidic interfaces, this approach enables the co-culture of multiple organoids derived from different tissues. For instance, when investigating drug absorption and metabolism in the digestive system, the organ-on-a-chip approach enables the co-culture of liver, stomach and intestinal organoids in interconnected microchip wells (Jin et al., 2018), modelling multi-organ pharmacokinetics and inter-tissue signalling. Accordingly, Zhu et al. generated cortical, hippocampal and thalamic brain assembloids by using a multilayered microfluidic chip with fluid flow, which allowed the fusion of organoids in specific patterns and enabled neural migration across regions, modelling inter-region communications (Zhu et al., 2023). Although Zhu et al. did not include vasculature in their study, their microfluidics platform has the potential to create vascularised, multi-region brain assembloids with fluid flow by using microfluidics. The main advantage of the organ-on-a-chip approach compared to other discussed approaches is its ability to provide physiologically relevant fluid flow – resulting in more homogeneous delivery of oxygen and nutrients – and recapitulate tissue microenvironments, which could aid the growth and maturation of complex organoid systems.

However, the design of microfluidic platforms requires specialised knowledge, such as laminar flow physics, fluid dynamics and computer-aided design, as well as custom engineering from device materials to modular components (Chiu et al., 2017). Clean room facilities (Rodríguez et al., 2023) and high equipment costs, such as syringe pumps (US$350–3500; Park and Shin, 2024) and pressure pumps (∼US$6000; Gao et al., 2020), create barriers for researchers who are considering microfluidic platforms. Although commercial chips are available, chips from different suppliers are often incompatible; and preferences for custom designs cause protocol variability, which hinders the comparison of experimental results across groups (Ortseifen et al., 2020). To bridge these gaps, interdisciplinary collaboration between microfluidics engineers and biotechnologists, as well as standardisation of protocols, is essential to achieve powerful high-throughput platforms for organoid research.

## Current challenges and future directions in organoid disease modelling

Current challenges in the field of vascularised organoids for disease modelling focus on developing mature, functional and perfusable vascular networks that closely mimic the structure and physiology of human tissues. Many established organoid systems lack vasculature, and those that successfully incorporate blood vessels often do not have full perfusability or the characteristics of native vessels. Microfluidics might be a way to improve perfusion by providing fluid flow and shear stress, creating a more dynamic microenvironment. But these methods often involve complex fabrication processes, such as laminar flow physics and fluid dynamics design with computer-aided design software. Costly equipment and custom designs also impede the standardisation and reproducibility across laboratories, hindering the production of organoids at a clinically relevant scale. Interdisciplinary collaboration between engineers and biologists is crucial for standardising physiologically relevant hemodynamics to model various diseases (see poster ‘Future directions’).

Currently, the majority of vascularised organoid models of diseases still lack immune components (Miao et al., 2025), which limits their application because immuno−vascular interactions are critical in the progression of many diseases – e.g. cardiovascular diseases (Wang et al., 2025), liver fibrosis (Kostallari et al., 2025) and autoimmune diseases (Porsch and Binder, 2024). Introduction of immune components, such as macrophages, B-and T-cells, may enhance the physiological and clinical relevance of organoid-based models (see poster ‘Future directions’). Furthermore, utilising patient-derived cells can allow personalised disease modelling to predict disease risks, disease progression and drug responses. By combining parenchymal, endothelial, mural and immune cells in a patient-specific manner, vascularised-immune-organoids will pave the way for a new era of disease modelling.

With advancements in artificial intelligence and computational biology, automated production of vascularised organoids will be possible, establishing real-time feedback on organoid characteristics (e.g. size, improper aggregation of cells and presence of necrosis) and vasculature characteristics (e.g. angiogenesis, vessel rupture and/or leakage and vasodilation and/or vasoconstriction) to ensure healthy organoids (see poster ‘Future directions’). This can help researchers characterise organoids and vascular networks in 3D, allowing them to adjust culture conditions, such as fluid flow, oxygen levels and medium composition at an early stage, improving consistency and efficiency of organoid production, as well as reducing resource wastage due to the creation of undesirable organoids. Similarly, artificial intelligence-driven imaging, data analytics and sensor analysis can predict organoid developmental trajectories, identify off-target morphogenesis and recommend real-time adjustments. While each fabrication method of vascularised organoids has its distinct advantages and limitations, continuing efforts in the field will expand its applicability and aid in understanding complex vascular pathologies. Collectively, these advances will enhance the physiological relevance, scalability and reproducibility of vascularised organoids, paving the way for their utilisation in precision medicine, drug development and regenerative therapies.

## Poster

Poster
