# Supplementary figures and images for: Disease modelling with in vitro vascularised organoids
Source: Dis Model Mech. 2026 May 20;19(6):dmm052769. doi: 10.1242/dmm.052769 (PMC13225715; doi:10.1242/dmm.052769)

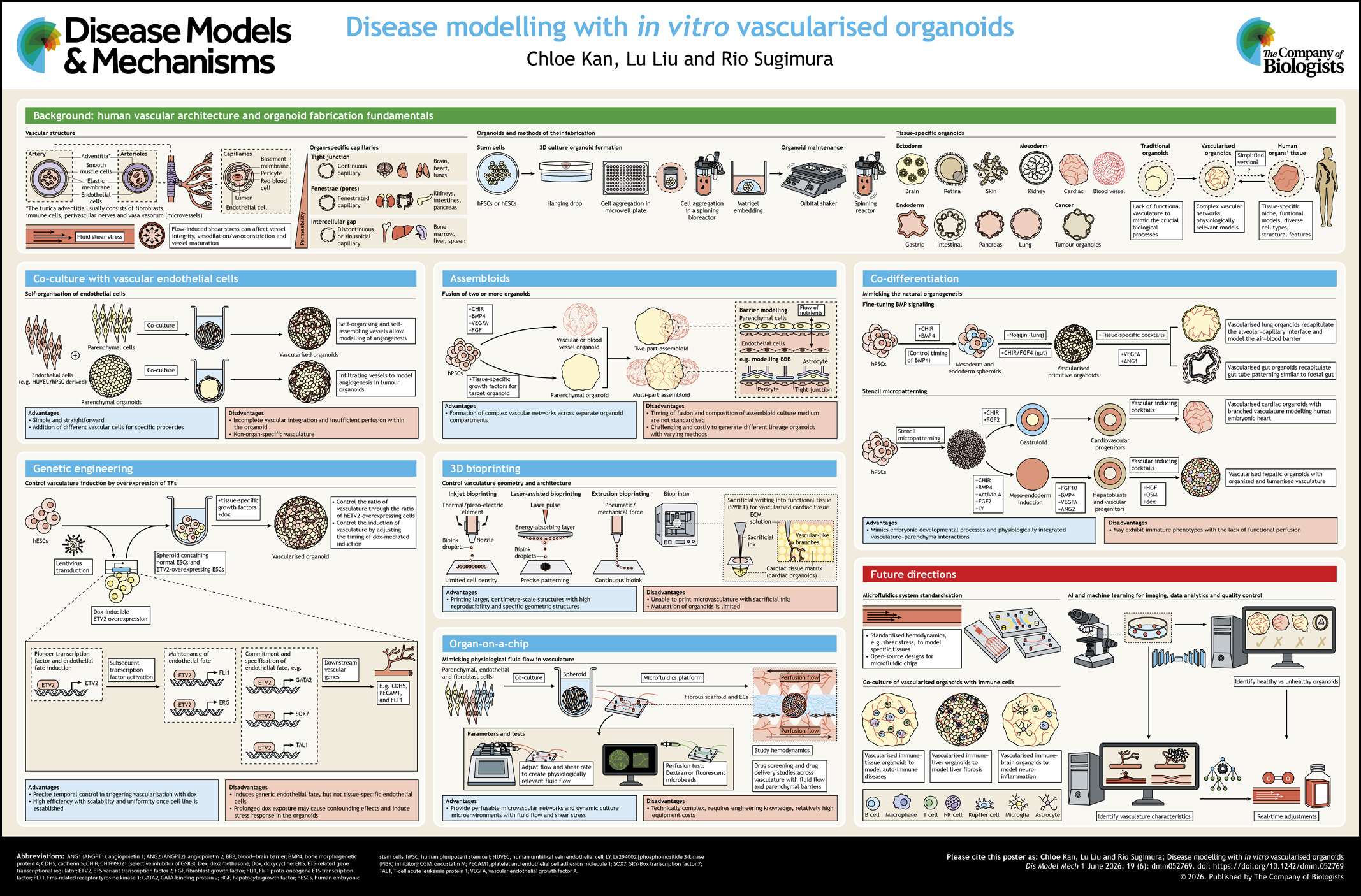

Supplement: Poster [file dmm-19-052769-s1.jpg]
